# From Dyspnea to Diagnosis: Neutrophilic Pleural Effusion With Elevated Adenosine Deaminase As the First Clue to New-Onset Rheumatoid Arthritis

**DOI:** 10.7759/cureus.101247

**Published:** 2026-01-10

**Authors:** Syed A Moosa, Lily Zonnoor, Amauri Gomez, Eugeniya Golub, Aleksander Feoktistov

**Affiliations:** 1 Rheumatology, Kings County Hospital, Brooklyn, USA; 2 Rheumatology, State University of New York Downstate Medical Center, Brooklyn, USA; 3 Internal Medicine, St. John's Episcopal Hospital, Far Rockaway, USA

**Keywords:** corticosteroids, interstitial lung disease, methotrexate, pleural effusion, rheumatoid arthritis, rheumatoid pleuritis

## Abstract

Rheumatoid arthritis (RA) rarely announces itself with a pleural effusion, yet when it does, the biochemical and pleural fluid profile often leads to a suspicion of infectious etiology at first. We describe a 44‑year‑old woman whose initial manifestation of seropositive RA was a large, unilateral, neutrophil‑rich, adenosine‑deaminase (ADA)-positive pleural effusion. The case draws attention to a diagnostic pitfall: elevated pleural fluid ADA-although classically associated with tuberculous pleuritis-can also occur in rheumatoid pleuritis, highlighting that ADA levels must be interpreted in the full clinical context and should not, by themselves, prompt initiation of anti-tuberculous therapy. By combining serial fluid analysis with targeted serology, we avoided unnecessary antimycobacterial therapy, initiated immunosuppression early, and achieved complete radiographic resolution. The report emphasizes the need to identify extra-articular conditions (especially pleural involvement) as potential early indicators of RA development.

## Introduction

Rheumatoid arthritis (RA) represents a systemic autoimmune condition that mainly targets synovial joints [1]. Extra-articular manifestations, including pleural effusions, occur in a minority of patients - often associated with long-standing disease [2,3]. A literature review article in 2007, however, documents effusions arising months or even years before overt joint inflammation, challenging the traditional timeline [4]. During this time, diagnostic confusion is common because rheumatoid fluid chemistry (very low glucose, low pH, high LDH) overlaps with that of complicated parapneumonic effusions, while ADA values that exceed 40 U/L usually steer clinicians toward tuberculosis [5]. Early-phase rheumatoid effusions may also show neutrophil predominance before transitioning to lymphocytic predominance, further complicating the clinical picture [4]. Here, we present a case that highlights these issues and illustrates how serial thoracenteses and thoughtful serological testing can clarify the picture.

## Case presentation

**Figure 1:**
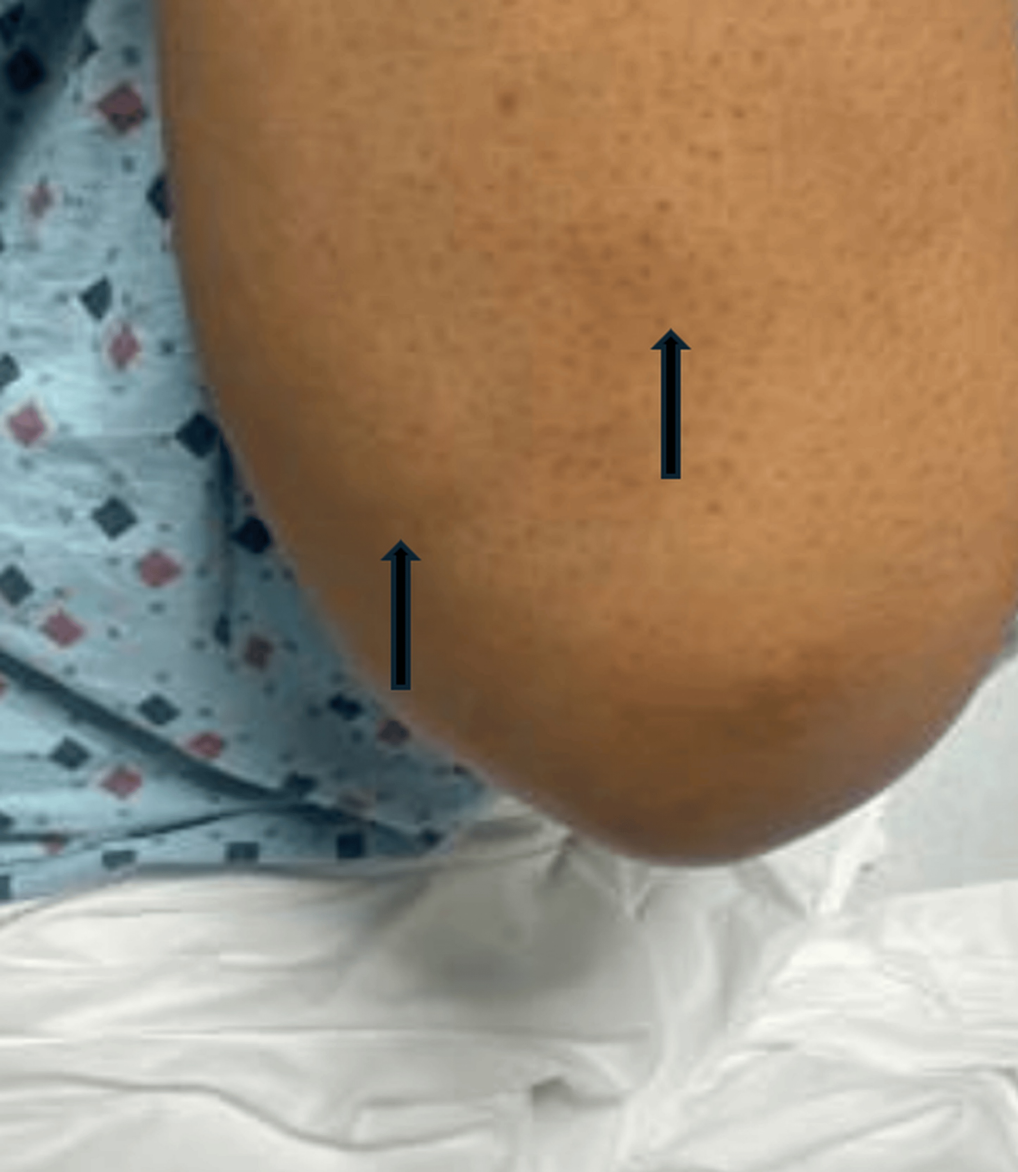
Subcutaneous nodules on the elbows – extensor surface (black arrows).

A 44‑year‑old woman with no significant past medical history presented in December 2024 with a two‑week history of progressively worsening dyspnea. Closer questioning uncovered six weeks of polyarthralgia involving both wrists, hands, and ankles, which had not impacted her daily activities. She denied fever, chills, weight loss, or recent travel, and had no known tuberculosis risk factors (no household or occupational exposure, no prior latent‐TB infection, and no immunosuppression). Examination revealed tachycardia, diminished breath sounds over the left lung base, asymmetrical swelling and tenderness of the metacarpophalangeal and proximal interphalangeal joints (synovitis), and firm subcutaneous nodules on the extensor surfaces of both elbows (Figure 1).

The patient displayed none of the clinical signs that indicate systemic lupus erythematosus (SLE) or mixed connective tissue disease (MCTD), including malar rash, oral ulcers, Raynaud's phenomenon, or myositis.

**Figure 2:**
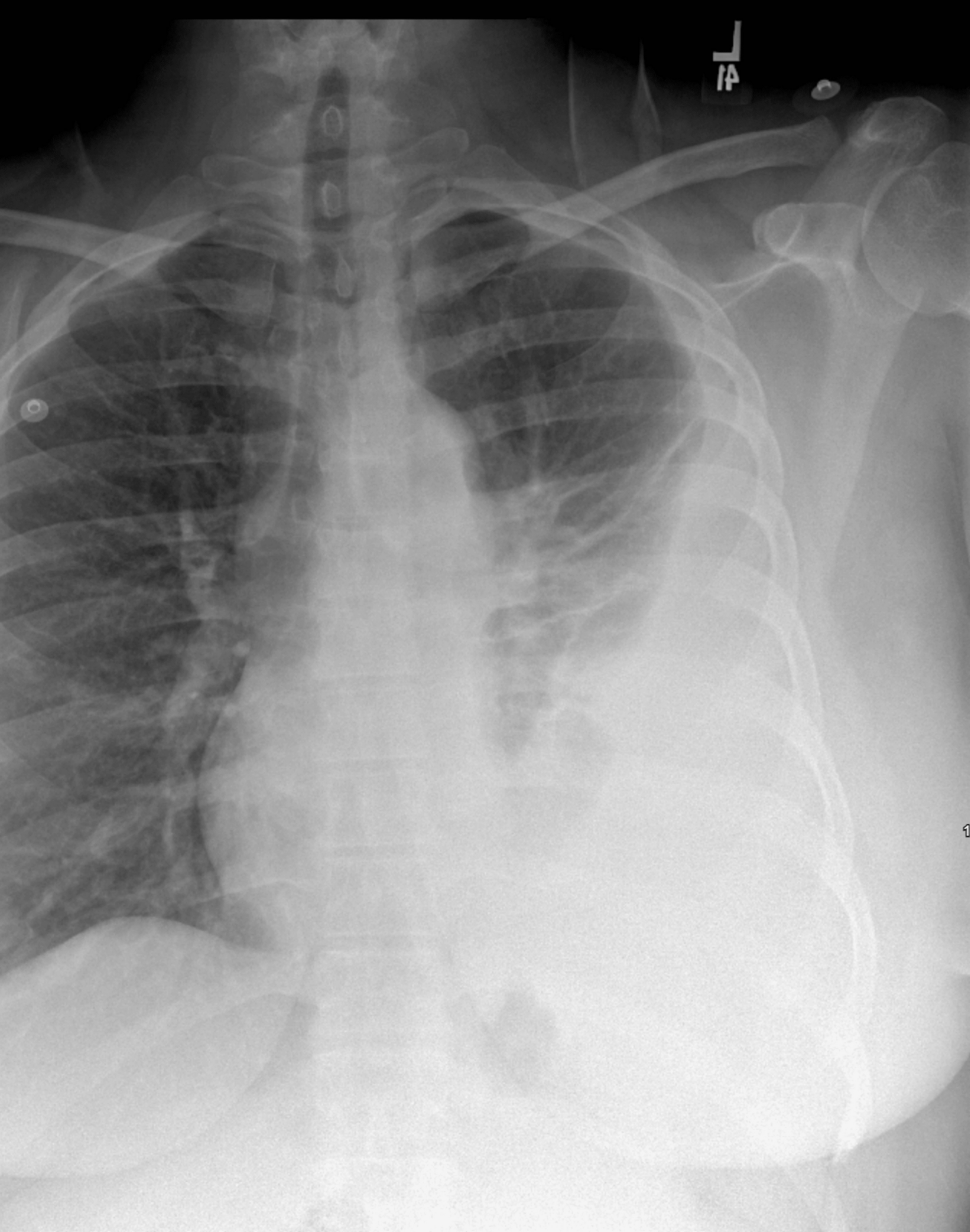
Chest X-ray showing left lower lobe pleural effusion with subjacent atelectasis.

**Figure 3:**
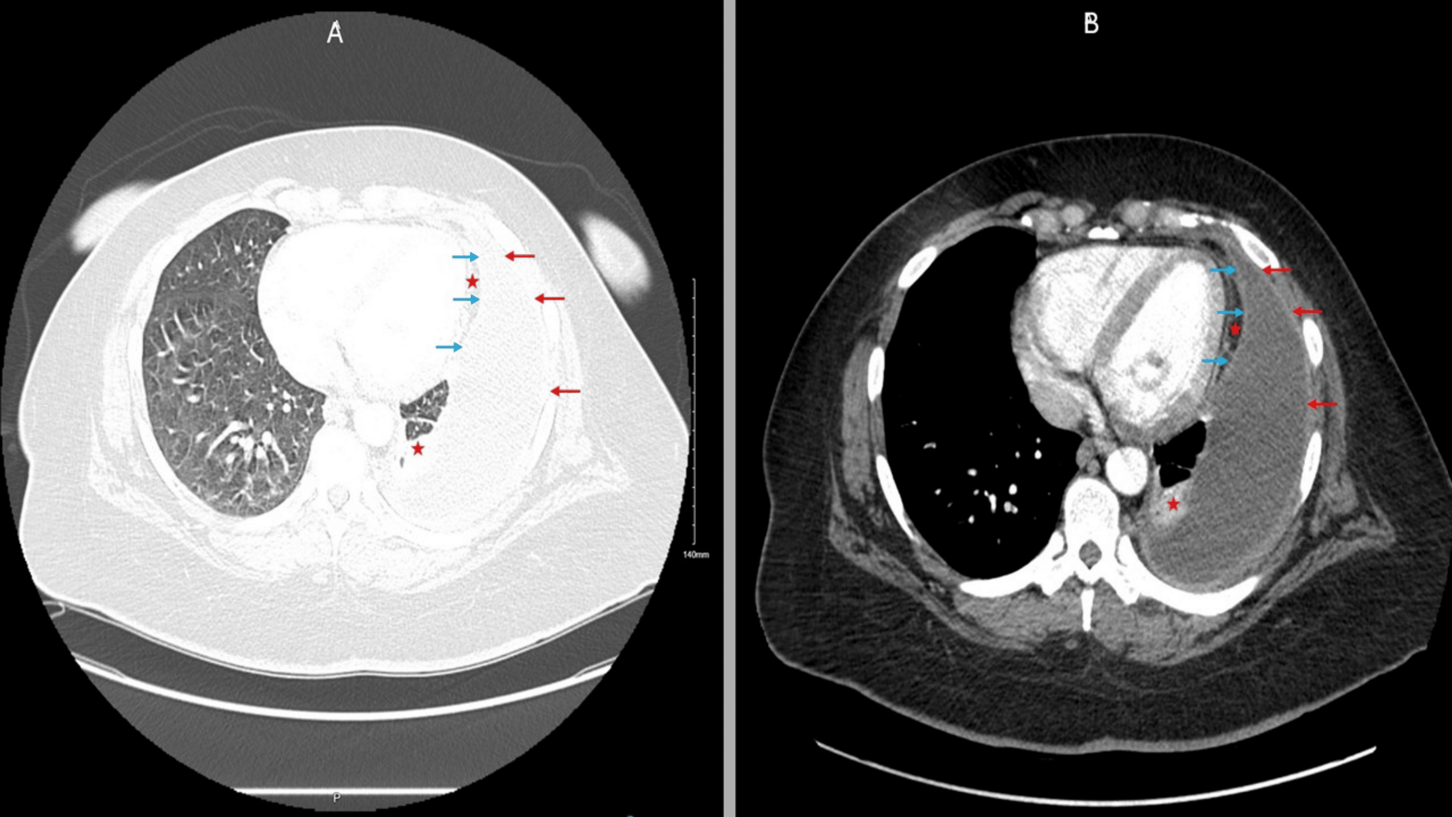
CT angiogram demonstrating split pleura sign in a large left pleural effusion (lung-window and standard views). No pulmonary embolism detected. Blue arrows: Thickened visceral pleura; Red arrows: Thickened parietal pleura; Red star: Compressive atelectasis. Panel A: Lung-window reconstruction. Panel B: Standard contrast-enhanced CTA view. Abbreviation: CTA = computed tomographic angiography.

Initial laboratory investigations performed before the rheumatology consultation revealed leukocytosis (12.66 × 10³ cells/µL), thrombocytosis, and high levels of C‑reactive protein (CRP; 197.9 mg/L) and erythrocyte sedimentation rate (ESR; 95 mm/h) along with a normal procalcitonin level. D‑dimer was 5,462 ng/mL. Despite a positive ANA test result at 1:160 showing a speckled pattern, the anti-double-stranded DNA and complement components C3 and C4 displayed levels within normal limits. The chest contrast‑enhanced computed tomography angiography (CTA), performed due to significantly elevated D-dimer, showed a large loculated left pleural effusion with a split‑pleura sign (highly suggestive of empyema or complicated parapneumonic effusion) next to adjacent atelectasis (Figures 2-3).

A chest tube was placed by the pulmonology team. Thoracentesis revealed a hazy, yellow exudative fluid with glucose 18 mg/dL, LDH 3,561 U/L and total protein 5.2 g/dL. Light’s criteria confirmed exudate as shown in Table 1.

**Table 1:** Light's criteria–exudate determination Abbreviation: LDH: Lactate dehydrogenase

Light’s Criterion	Calculation	Value	Meets Exudate Threshold?
Pleural fluid protein / Serum protein	5.2 / 8.3	0.63	Yes (≥ 0.50)
Pleural fluid LDH / Serum LDH	3561 / 322	11.06	Yes (≥ 0.60)
Pleural fluid LDH > ⅔ upper-limit normal serum LDH (214 U/L)	3561 > 142.7	—	Yes

The pleural fluid cytology and microbiological results were pending. The articular findings, combined with subcutaneous nodules and fluid chemistry analysis, led to a strong suspicion of rheumatoid arthritis (RA). The laboratory tests ordered included rheumatoid factor (RF), anti‑cyclic citrullinated peptide antibody (anti‑CCP), and an extended autoimmune panel (anti‑RNP, anti‑Sm, anti‑SSA/SSB) to assess potential overlap syndromes. 

High‑titer RF (119 IU/mL) and anti‑CCP (379 U/mL) returned first. With a normal procalcitonin level, infection appeared less likely; therefore, prednisone 30 mg daily, methotrexate 15 mg weekly, and folic acid 1 mg daily were initiated.

Subsequent result of complete pleural‑fluid analysis showed neutrophil predominance (64 %) and adenosine deaminase (ADA) 57 U/L (in the setting of "split pleura sign" which raises concern of possible complicated parapneumonic effusion or empyema), prompting the primary and pulmonary teams to broaden antibiotics and consider tuberculosis. Infectious‑disease specialists were consulted, and video‑assisted thoracoscopic surgery (VATS) with pleural biopsy was discussed. The remainder of the autoimmune panel was unremarkable except for mildly positive anti‑RNP antibodies. Although cultures had not yet resulted, serum procalcitonin was normal, and we recognized that rheumatoid pleural effusions can present with an early neutrophil predominance and occasionally elevated ADA, especially in the setting of the patient's subcutaneous nodules and synovitis on physical exam. On that basis (expanded in the Discussion section), we maintained our initial suspicion that RA-related pleuritis is the leading diagnosis. Coordinating with pulmonology, a repeat thoracentesis one week later revealed a shift to lymphocyte predominance (54 %), supporting our impression. 

A summary of the pertinent labs of the patient is presented in Table 2, and a comparison of the interval pleural fluid analysis is presented in Table 3.

**Table 2:** Summary of key laboratory tests (December 2024) Abbreviations: WBC: White Blood Cell Count; RBC: Red Blood Cell Count; Hgb: Hemoglobin; Hct: Hematocrit; RDW: Red Cell Distribution Width; CMP: Comprehensive Metabolic Panel; BUN: Blood Urea Nitrogen; CRP: C-Reactive Protein; ESR: Erythrocyte Sedimentation Rate; LDH: Lactate Dehydrogenase; RF: Rheumatoid Factor; Anti-CCP: Anti-Cyclic Citrullinated Peptide; ANA: Antinuclear Antibody; anti-dsDNA: Anti–Double-Stranded DNA; C3/C4: Complement Component 3/4; Jo-1: Anti-Jo-1 Antibody; RNP: Ribonucleoprotein; SS-A/SS-B: Sjögren's Syndrome Antibodies A/B; Sm: Anti-Smith Antibody; Scl-70: Anti–Scleroderma-70 Antibody; AI: Antibody Index

Initial Tests (before Rheumatology Consult Request)	Value	Reference Range	Units
WBC (initial lab)	12.66	4.50 - 10.90	K/uL
Hemoglobin (initial lab)	10.0	12.0 - 16.0	g/dL
Hematocrit (initial lab)	31.9	37.0 - 47.0	%
Platelets (initial lab)	522	130 - 400	K/uL
Neutrophils (%)(initial lab)	76.5	38.7 - 60.3	%
ESR (initial lab)	95	0 - 20	mm/hr
CRP (initial lab)	197.9	1.00 - 4.00	mg/L
Procalcitonin (initial lab)	0.10	0.00 - 0.50	ng/mL
LDH (initial lab)	322	135 - 214	U/L
D-Dimer (initial lab)	5462	<=230	ng/mL
ANA Titer (initial lab)	1:160	<1:80	
ANA Pattern (initial lab)	Speckled		
dsDNA (initial lab)	<1	<=4	IU/mL
C3 (initial lab)	161	86 - 184	mg/dL
C4 (initial lab)	21	20 - 58	mg/dL
Subsequent Tests (after Rheumatology Consultation)	Value	Reference Range	Units
RF (obtained after rheumatology consultation)	119	<14	IU/mL
Anti-CCP (obtained after rheumatology consultation)	379	<=16.9	U/mL
Jo-1	<0.2	<=0.9	AI
RNP	1.3	<=0.9	AI
SS-A	<0.2	<=0.9	AI
SS-B	<0.2	<=0.9	AI
Sm	<0.2	<=0.9	AI
Scl-70	<0.2	<=0.9	AI

**Table 3:** Pleural fluid analysis WBC: White Blood Cell Count; LDH: Lactate Dehydrogenase

Parameter	12/13/2024	12/19/2024	Units
Appearance	Hazy	Cloudy	
Color	Yellow	Straw	
WBC	3,276	11,170	Cells/uL
Neutrophils (%)	64	25	%
Lymphocytes (%)	10	54	%
Glucose	18	18	mg/dL
LDH	3,561	—	U/L
Protein	5.2	—	g/dL
Albumin	2.4	—	g/dL
Adenosine Deaminase	57 (normal range: 0-30)	—	U/L

Cultures (including acid‑fast bacilli and QuantiFERON‑TB Gold) and cytology were negative. With infectious and malignant causes excluded, all teams agreed the effusion was rheumatoid. Prednisone 30 mg daily and methotrexate were continued, with methotrexate titrated to 25 mg weekly. Dyspnea and joint pain improved rapidly; the pigtail catheter was removed after two weeks. Follow‑up imaging demonstrated near‑complete resolution of the effusion, and complete radiographic clearance was confirmed at three months.

By April 2025, the patient had attended three outpatient rheumatology visits. Her disease remained in remission (Disease Activity Score‑28 using CRP = 2.20). The subcutaneous nodules had also disappeared by then. She continues methotrexate 25 mg weekly and folic acid 1 mg daily, with near-complete tapering off of prednisone. She is also being closely monitored for a potential RA-SLE overlap (Rhupus) at an early stage in the context of positive RNP.

## Discussion

The constellation of polyarthritis (synovitis), subcutaneous nodules, high rheumatoid factor and anti-CCP titres, and a neutrophil-to lymphocyte-predominant pleural exudate, and remarkable clinical improvement after receiving methotrexate most strongly supported seropositive rheumatoid arthritis (RA). Her extended autoimmune panel showed a low-positive anti-RNP (1.3 AI), while anti-dsDNA, anti-Sm, and anti-SSA/SSB remained negative, and complement levels were normal. A speckled ANA at 1:160 is nonspecific and is also reported in patients with RA [6]. In the absence of clinical features typical of systemic lupus erythematosus or mixed connective tissue disease, such as photosensitive rash, oral ulcers, Raynaud phenomenon, proximal muscle weakness, hardened, thickened skin changes affecting the distal extremities (acroscelrosis), sclerodactyly, features of interstitial lung disease, or esophageal dysmotility, the presentation was classified as RA-associated pleuritis.

In the pre‑biologic era, rheumatoid pleuritis clustered in older men with long‑standing, nodular disease [7]. Contemporary reports, including our patient, show a broader demographic: seropositive women in their fourth and fifth decades whose articular complaints are mild or clinically silent [7]. Among 456 high‑ADA effusions collected in a modern multicenter study, autoimmune conditions (predominantly RA) accounted for roughly 3% of cases, matching lymphoma and surpassed only by tuberculosis and empyema [5].

Immune‑complex deposition on the pleural mesothelium activates complement, recruits neutrophils, and drives the canonical pattern of very low glucose, low pH, and high LDH [8]. High ADA levels in pleural effusions reflect intense T-cell-mediated immunity-not a disease-specific signal- particularly T-cell and monocyte/macrophage activation, but are not specific to rapid T-cell turnover or to tuberculous pleuritis alone [5,7]. Experimental work has demonstrated a direct correlation between T‑lymphocyte density and ADA activity, explaining why values as high as 110 U/L have been documented in rheumatoid effusions [9].

Although ADA > 40-50 U/L often triggers anti-tubercular therapy, there is no threshold that reliably separates tuberculosis from rheumatoid pleuritis [5]. Clinical context and serial fluid analysis are therefore paramount. In our patient, infection was unlikely: cultures remained sterile, and procalcitonin was normal.

Within the first week, neutrophils dominate, giving the cytology an empyema-like appearance; by days 7-11, lymphocytes overtake neutrophils as acute inflammation subsides [4]. Our patient followed the same trajectory, reinforcing the benign, self-evolving nature of the process once infection is excluded.

Management

Timely immunosuppression is the cornerstone once infection has been ruled out. Corticosteroids accelerate resolution and forestall fibrous peel or trapped lung physiology, while methotrexate provides durable control [7]. There is a lack of high-quality, direct evidence specifically for the management of recurrent, steroid-refractory rheumatoid pleural effusion, and most recommendations are extrapolated from guidelines for rheumatoid arthritis-associated interstitial lung disease; further research is needed to define optimal therapy [8-10]. Large or loculated collections threatening ventilation warrant tube drainage; chemical pleurodesis or decortication is rarely necessary when systemic inflammation is controlled [7]. Our patient responded briskly to prednisone and methotrexate, with complete radiographic clearance and sustained remission at six-month follow-up. High anti-CCP titers, as seen in this patient, are associated with increased risk for interstitial lung disease (ILD), warranting longitudinal monitoring [2,11]. Given the isolated anti-RNP reactivity, the patient will be followed every six months with repeat serologies and complement levels, and instructed to report any new cutaneous, mucosal, or vascular symptoms promptly to detect a potential RA-SLE overlap (Rhupus) at an early stage.

Clinical implications

Elevated ADA with low glucose warrants a broad differential diagnosis rather than reflex diagnosis and management of infections, especially tuberculosis. Integrating fluid chemistry, its temporal evolution, and autoimmune serology prevents unwarranted antimicrobials and allows early disease-modifying therapy. Vigilant respiratory surveillance remains important, as RA-related lung disease still carries excess mortality [8].

## Conclusions

Pleural effusion may herald the first sign of RA. ADA values often considered suggestive of an infectious process, such as tuberculosis, can occur in rheumatoid pleuritis, particularly during the neutrophil‑rich early phase. Serial fluid analysis, coupled with targeted serology, enables timely immunosuppression and averts pleuro‑pulmonary complications.
